# Repeated MDA5 Gene Loss in Birds: An Evolutionary Perspective

**DOI:** 10.3390/v13112131

**Published:** 2021-10-22

**Authors:** Veronika Krchlíková, Tomáš Hron, Martin Těšický, Tao Li, Jiří Hejnar, Michal Vinkler, Daniel Elleder

**Affiliations:** 1 Institute of Molecular Genetics of the Czech Academy of Sciences, 14220 Prague, Czech Republic; veronika.krchlikova@img.cas.cz (V.K.); tomas.hron@img.cas.cz (T.H.); jiri.hejnar@img.cas.cz (J.H.); 2 Department of Zoology, Faculty of Science, Charles University, 12843 Prague, Czech Republic; martin.tesicky@natur.cuni.cz (M.T.); tao.li@natur.cuni.cz (T.L.)

**Keywords:** avian genome, viral sensors, innate immunity, gene loss

## Abstract

Two key cytosolic receptors belonging to the retinoic acid-inducible gene I (RIG-I)-like receptor (RLR) family sense the viral RNA-derived danger signals: RIG-I and melanoma differentiation-associated protein 5 (MDA5). Their activation establishes an antiviral state by downstream signaling that ultimately activates interferon-stimulated genes (ISGs). While in rare cases *RIG-I* gene loss has been detected in mammalian and avian species, most notably in the chicken, *MDA5* pseudogenization has only been detected once in mammals. We have screened over a hundred publicly available avian genome sequences and describe an independent disruption of *MDA5* in two unrelated avian lineages, the storks (Ciconiiformes) and the rallids (Gruiformes). The results of our RELAX analysis confirmed the absence of negative selection in the *MDA5* pseudogene. In contrast to our prediction, we have shown, using multiple dN/dS-based approaches, that the *MDA5* loss does not appear to have resulted in any compensatory evolution in the *RIG-I* gene, which may partially share its ligand-binding specificity. Together, our results indicate that the *MDA5* pseudogenization may have important functional effects on immune responsiveness in these two avian clades.

## 1. Introduction

Genome evolution in vertebrates is strongly influenced by their pathogens, including viruses [1,2]. Long-term interactions between hosts and infectious agents result in continuous reciprocal adaptations optimizing the host’s immune defense machinery. In general, adaptive changes in host gene sequences are commonly manifested as an increase of the dN/dS ratio (ratio of nonsynonymous over synonymous substitution rates). Such positive—diversifying—selection is the hallmark of the evolutionary arms’ race between pathogens and hosts. Other types of adaptive changes include gene gains (e.g., the generation of multigene families by gene duplication) and gene losses [3,4].

Avian viruses include important pathogens with an impact on human health due to cross-species transmission events, best exemplified by the avian influenza viruses (AIV). Here, we focus on avian RIG-I-like receptors (RLR), a group of pattern-recognition receptors for RNA viruses (reviewed in [5]). RLRs can recognize intracellular viral RNA and mediate the transcriptional induction of type I interferons and other relevant genes, leading to the establishment of an antiviral host response. The RLR family consists of three members: retinoic acid-inducible gene I (RIG-I) [6], melanoma differentiation-associated gene 5 (MDA5) [7] and laboratory of genetics and physiology 2 (LGP2) [8]. All RLRs have a central helicase domain and a carboxy-terminal domain (CTD), which are directly involved in the binding of foreign RNA molecules. RIG-I and MDA5 also contain two amino-terminal caspase activation and recruitment domains (CARDs), which initiate signaling transduction. In contrast to this, LGP2 is not capable of mediating downstream signaling and its assumed role is to regulate the other two RLR sensors [9]. LGP2 was also shown to inhibit Dicer-dependent processing of long double-stranded RNAs [10]. RIG-I and MDA5 are specialized in the detection of diverse RNA structures, and their roles in virus sensing are mostly non-overlapping [11,12,13]. While RIG-I recognizes short RNA ligands with 5′-phosphorylated ends, MDA5 detects long RNAs, including viral replication intermediates. Both RIG-I and MDA5 coding sequences are variable at interspecific level. There is evidence showing that mammalian RLRs are subjected to strong positive selection [14,15]. Although site-specific positive selection may alter receptor function, especially in cases when the positively selected sites (PSS) overlap with the functional sites involved in viral RNA binding, even more profound phenotypic effects can be expected in cases of gene loss.

Importantly, chicken, the key avian research model species, lacks *RIG-I*, which is supposed to contribute to its high susceptibility to AIV [16,17]. It is still not fully understood to what degree chicken cells are defective in recognizing certain foreign RNA structures due to the *RIG-I* loss. Compensatory changes have been described that have developed in chicken *MDA5* and can at least partially rescue the compromised viral RNA recognition [18,19].

The availability of a large number of vertebrate genome sequences has created an opportunity to investigate the molecular evolution of immune genes more broadly and in non-model organisms. One recent study showed that avian RLRs evolved under positive selection [20]. It also reported the absence of *RIG-I* in several additional avian species besides chicken.

In this study, we screened over a hundred avian genomes presently available in the public sequence databases and analyzed the *MDA5* and *RIG-I* sequences obtained. Having determined the absence of the full-length coding *MDA5* sequence in some of the analyzed avian genomes, we checked for the phylogenetic pattern of the *MDA5* loss in birds and mapped its evolutionary history. As the compensatory evolution of vertebrate RLRs has been proposed in several cases, we also determined the selection patterns in *RIG-I*. Assuming the functional importance of positive selection acting on the *RIG-I*, we also linked the detected variation at PSSs with their topological position in the predicted protein 3D structure, determining the site-specific accessibility to ligands and comparing our data with functional evidence reported in the current literature.

## 2. Methods

### 2.1. Sequence Datasets

Avian *RIG-I* and *MDA5* coding sequences were obtained either from the NCBI assembled genome database or de novo identified by screening and assembling of the raw sequencing data from the NCBI SRA database. This was carried out using NCBI Blastn searches [21,22] and targeted assembly in DNASTAR Lasergene software. To analyze the selection pressure acting on the *RIG-I* sequences in a context of presence/absence of the *MDA5* gene, we used a dataset of *RIG-I* and *MDA5* sequences from 101 avian species where we were able to assemble a full length coding sequence for both genes. All coding sequences in FASTA format are provided in Figure S1, and the accession numbers of genomic contigs and SRA datasets in Figure S2. Evolutionary relationships between individual species were extracted from BirdTree.org. Specifically, a phylogenetic species tree was generated using the BirdTree tool [23,24] based on the Ericson all species dataset. Seventeen species from our dataset that were missing in the Ericson dataset were substituted by their closest relatives, mostly within the same genus level, which should not alter the tree topology.

### 2.2. Analysis of Selection Relaxation

Intact nucleotide coding sequences of nonfunctional *MDA5* orthologs were reconstructed—frameshift mutations were corrected and premature stop codons were masked. All *MDA5* sequences were then translated to amino acid sequences, aligned using the MAFFT tool [25] with default parameters, and converted back to the nucleotides. Final alignment was manually inspected and edited (Figure S3). To test that natural selection has been relaxed in putatively defected *MDA5* orthologs, we used the RELAX tool [26] implemented in the HyPhy v.2.5.25 package [27] with default parameters. All internal and external branches leading from the assumed points of the *MDA5* inactivation were marked as test branches. The rest of the phylogeny was marked as a reference set.

### 2.3. Positive Selection Analysis

Nucleotide coding sequences of avian *RIG-I* were translated to amino acid sequences, aligned using the MAFFT tool [25] with default parameters, and converted back to the nucleotides. Final alignment was manually inspected and edited (Figure S4).

Positive selection acting on avian *RIG-I* was evaluated using the PAML 4.7 package [28]. The model assuming several groups of residues specified by different dN/dS for each branch was employed (branch-site model). Presence of positive selection acting on particular branches was tested with the branch-site model of positive selection in the codeml program [29]. Specifically, modified branch-site model A (model = 2 NSsites = 2) was compared with the null model (dN/dS value fixed to 1) using the likelihood ratio Ttest (LRT). P values corresponding to positive selection significance were calculated based on LRT statistics.

The FEL, FUBAR [29,30] and MEME method [29,30,31] implemented in the HyPhy package were used to detect positively selected residues in the RIG-I protein sequence. FEL and FUBAR assume that the selection pressure for each site is constant along the entire phylogeny. MEME is a more specialized codon-based method to detect episodic selection pressure. P values representing significance of positive selection for each site were calculated using LRT statistics. Additionally, as an alternative to FEL, Bayes Empirical Bayes (BEB) positive selection analysis [32] implemented in PAML 4.7, was performed under the M2a site model.

### 2.4. Annotation of Positively Selected Sites

To minimize false positives, only highly supported positively selected sites (PSS)—identified based on the consensus of at least three selection methods used—were considered in the following analyses. The key physicochemical properties (molecular charge, hydrophobicity, physicochemical property groups based on the [33]) of PSSs were identified and mapped onto the linear amino acid sequences using the Weblogo 3 application [34]. PSSs were also plotted on RIG-I domain structure according to [35] and visualized by DOG, v. 1.0 [36]. List of PSSs was also compared with previously described RIG-I functional sites and with previously described PSSs in birds and other organisms (Figure S5). The proximity of detected PSSs to previously reported functional sites was evaluated. The minimal distance considered as a close proximity to the functional site was 5Å.

### 2.5. Structural Analysis

Since it has been shown that RIG-I undergoes distinct conformational changes when RNA is bound [35], we performed the structural analysis of avian RIG-I with both “open” (without ligand bound) and “closed” (with RNA bound) structure.

Although the duck Protein Data Bank (PDB) structure exists, it lacks some parts containing predicted PSSs. 3D modelling of duck open RIG-I structure was therefore performed using I-TASSER [37] with duck crystal structure (PDB ID: 4A2W) as a template. The best structural model was selected based on the C-score. The quality of the final model was further evaluated using the ModFOLD Model Quality Assessment Server v. 8 [38].

Identified PSSs were visualized together with previously described functionally relevant sites (Figure S4) in the PyMOL Molecular Graphics System (Version 2.0.7 Schrödinger, LLC) on 3D protein model of “open” conformation and also directly on duck RIG-I crystallographic structure of helicase domains with bound RNA (PDB ID 4A36). The distances between PSSs and functionally relevant sites were measured using function iterate implemented in PyMol. Consistent with [39], given the putative span of hydrogen bonds, salt bridges [40], and longer-range hydrophobic interactions [41], PSSs were considered in close topological proximity to the functional residues only if located < 5 Å apart.

To evaluate whether the PSSs are localized on the RIG-I surface, allowing their interaction with ligands, we calculated residue solvent exposure (solvent accessibility of a protein residue; RSA) for each site of the “open” and “closed” structure using the xssp web server [42]. RSA estimates for each PSS highly corresponded with both conformational states (Pearson’s correlation coefficient: r = 0.696; N = 47). For further analysis, we limited our scope mostly to surface accessible sites when RSA > 20.0%.

## 3. Results

### 3.1. Identification of MDA5 in Various Avian Species

In order to better understand the evolution of avian RLR genes, we first collected a comprehensive set of MDA5 coding sequences from various bird species. Besides the already annotated gene records in the National Center for Biotechnology Information (NCBI) databases, we also tried to de novo identify these genes by screening and assembling the raw sequencing data from the NCBI Short Read Archive (SRA). Interestingly, in this preliminary dataset, we found the disruption of the *MDA5* gene in nine species: *Ciconia boyciana*, *C. ciconia*, *C. episcopus*, *Porzana atra*, *Gallirallus okinawae*, *Heliornis fulica*, *Balearica regulorum*, *Antigone vipio* and *Grus nigricollis*.

**Figure 1:**
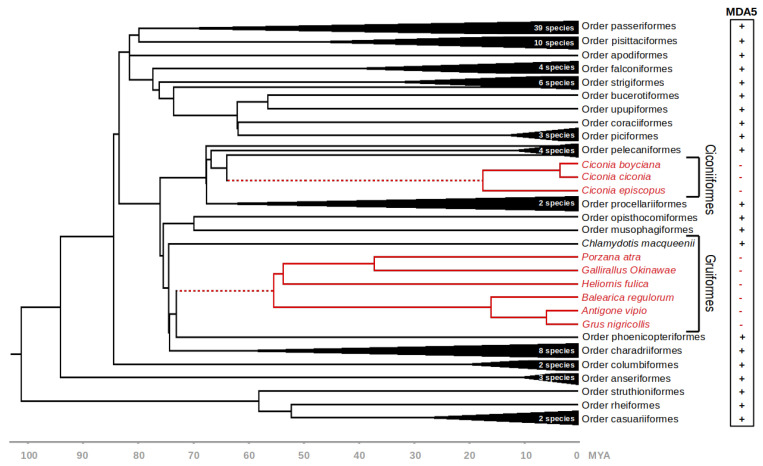
Presence or absence of functional *MDA5* gene in various avian species. Evolution of avian species analyzed in this study is shown by a chronogram obtained from BirdTree.org [23]. Orders Ciconiiformes and Gruiformes—where disruption of *MDA5* gene was observed—are shown in detail. Full red lines indicate a lineage with nonfunctional *MDA5* gene and dashed red lines indicate the predicted evolutionary time interval of the inactivation event. Time scale axis is shown below. MYA—million years ago.

For the following analyses, we used a dataset of *MDA5* coding sequences from 101 avian species (Figure S1). Where possible, the sequences were manually validated using publicly available RNAseq data deposited in the SRA. The dataset covered 22 avian orders, with one or more species representing each order. We then used BirdTree.org [23,24] database to indicate the time-calibrated phylogenetic relationships between individual species (Figure 1).

### 3.2. MDA5 Disruption in Ciconiiformes and Gruiformes

The phylogenetic position of the *MDA5*-lacking species suggests that the disruption of this gene occurred at least two times during the deep evolution of Neoaves. Based on the presence or absence of functional *MDA5* gene in closely related species, we estimate that the initial genetic inactivation in Ciconiiformes and Gruiformes occurred approximately 64.5–17.5 and 73.8–55.9 million years ago, respectively (Figure 1, dashed red line).

**Figure 2:**
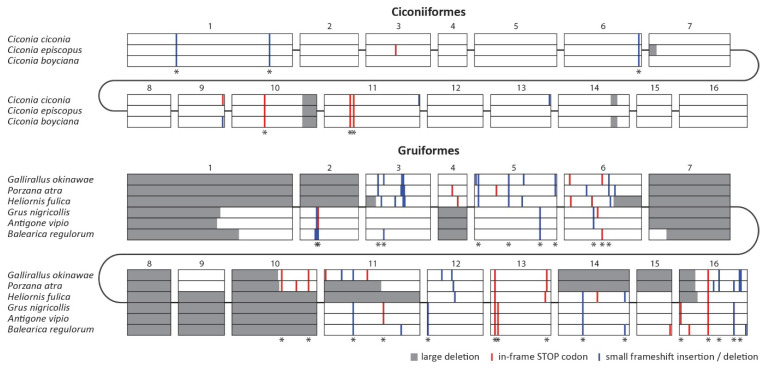
*MDA5* pseudogenization in the clades of Ciconiiformes and Gruiformes. Aligned *MDA5* coding sequences of all *MDA5*-disrupted species are shown schematically, separately for each order. Exons are represented in scale by open boxes. The predicted inactivating mutations are visualized as described in the figure legend. Mutations shared by at least two species within each order are marked by an asterisk.

To verify that the observed disruptions of the *MDA5* gene represent two independent ancestral events, we generated the nucleotide alignment of all disrupted *MDA5* coding sequences (Figure 2). Importantly, we were able to identify a number of shared inactivating mutations within each clade (Figure 2; sites with asterisk), but no changes were shared between the two groups, which strongly suggests parallel gene inactivation in the ancestors of these groups. The alignment also revealed the extent of inactivating mutations, which include in-frame stop codons, small frameshift indels and large deletions. In Ciconiiformes, there are approximately 10 inactivating mutations per sequence, of which more than half are shared by other species belonging to this order. In Gruiformes, we detected 20 to 30 inactivating mutations, of which the vast majority are shared by at least two species. The higher number of mutational events in Gruiformes is consistent with the estimated dating of the *MDA5* inactivation events, with gruiform *MDA5* pseudogenization predating the one in Ciconiiformes.

**Figure 3:**
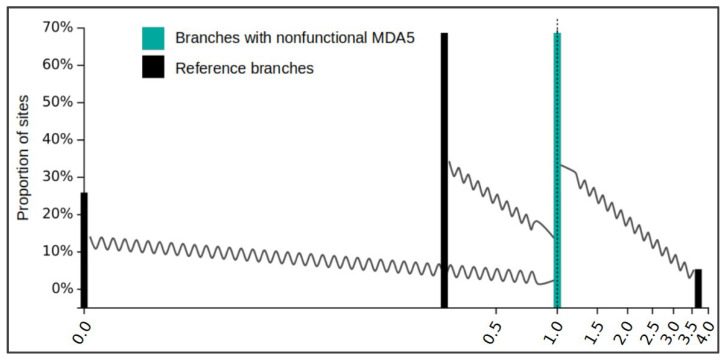
Relaxation of selection in nonfunctional *MDA5* avian orthologs. Result of the HyPhy RELAX analysis is shown as a diagram generated by the program. Black bars represent three categories of sites in functional *MDA5* avian orthologs with their dN/dS value estimates. Cyan bars (of the same dN/dS value for all three categories in this case), represent sites in disrupted *MDA5* avian orthologs described in this study. Shift of cyan bars to dN/dS value of 1 indicates relaxation of selection in tested species. The wavy lines indicate the shift in dN/dS values (for each category) between reference group and test group.

The disruption of a coding gene in an evolutionary lineage can be predicted to be followed by a relaxation of selection acting on the pseudogenized gene sequence. To confirm this for the avian *MDA5*, we used the RELAX analysis in the HyPhy software package. This analysis groups gene residues into categories defined by estimated dN/dS ratios—the indicator of selection pressure. The change in selection pressure strength is then tested by comparing a reference species group and a test species group. As a test group we used reading frame-corrected versions of all *MDA5*-disrupted orthologs where all stop mutations were masked out. The reference group contained sequences from the rest of the species in our dataset, with presumably intact *MDA5*. As we expected, the analysis showed a significant relaxation of negative selection in disrupted *MDA5* orthologs compared to sequences from other bird species with a *p* value < 0.001 (likelihood ratio test statistic = 69.42). The reference gene set comprised 25.92% of residues with dN/dS = 0.00 (very strong negative selection), 68.69% of residues with dN/dS = 0.26 (negative selection), and 5.38% of residues with dN/dS = 3.68 (diversifying selection), which can be considered a typical profile of a gene undergoing diversifying evolutionary selection (Figure 3, black bars). Compared to this, the test group with the inactivated *MDA5* gene has dN/dS for all residues equal to 1.00, which is a clear indication of a neutral evolution (Figure 3, cyan bars). This is consistent with the defective nature of *MDA5* in Ciconiiformes and Gruiformes.

### 3.3. No Evidence for Compensatory Evolution in Avian RIG-I in MDA5-Deficient Species

Since MDA5 and RIG-I recognize structurally similar ligands, we further asked whether the absence of functional MDA5 resulted in any compensatory evolution of the RIG-I receptor in the investigated avian lineages. To determine this, we collected the *RIG-I* full coding sequences for the species in our list in a way similar to what we had done for *MDA5* (Figure S1). Then we performed branch-site tests of *RIG-I* positive selection in the *MDA5*-deficient species and also in the other avian species in our dataset (Table 1). Our results indicate that despite significant positive selection acting in avian *RIG-I* (*p* value < 0.0001), the clades lacking functional *MDA5* do not show any increased selection strength in this gene (*p* value 0.0680). This did not confirm association of the positive selection acting on avian *RIG-I* with the *MDA5* loss.

**Figure 4:**
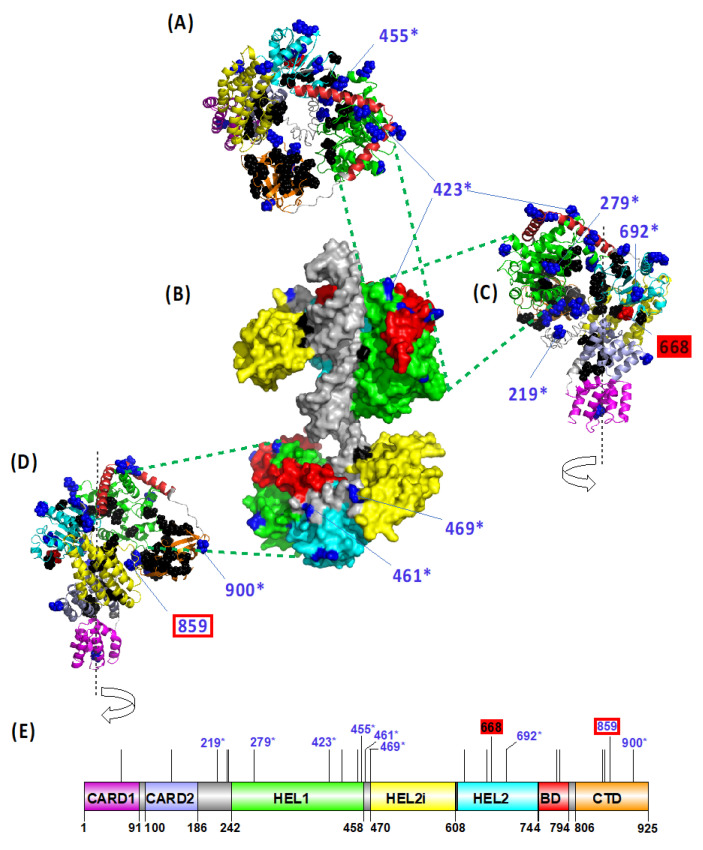
Topological distribution of the avian RIG-I positively selected sites (PSSs) in the protein structural model. Three-dimensional model of duck RIG-I is shown in its open state (ribbon models: (**A**)—top view, (**C**)—right-hand view, and (**D**)—left-hand view) and in a closed state with the ligand dsRNA bound (**B**). PSSs are also plotted on a linear scheme of a duck RIG-I domain structure (**E**). Previously reported functionally important residues are highlighted in black. PSSs are shown in blue. PSS in close proximity to the functional sites are highlighted by a red rectangle, and the ones that represent the functional sites are marked by a filled red box. Only PSSs being functional sites or located in close proximity to functional sites, or PSSs also identified in other studies (marked with an asterisk) are labelled by a site number according to the duck RIG-I CDS (GenBank ID EU363349.1). Different domains are shown in different colours: CARD1—N-terminal caspase activation and recruitment domain 1 (violet), CARD2—N-terminal caspase activation and recruitment domain 2 (light blue), HEL1—N-terminal RecA-like domain (green), HEL2i—insertion domain (yellow), HEL2—C-terminal RecA-like domain (cyanic blue), BD—bridging domain (red) and CTD—C-terminal domain (orange). For details regarding structure modelling and amino acid site annotations, see Methods section.

Finally, adopting four different methodological approaches (PAML, MEME, FEL and FUBAR), we targeted the positive selection acting in avian *RIG-I* at specific positively selected sites (PSS). In total, 22 PSSs in *RIG-I* were identified based on the consensus of at least three methods (Figure S5). To predict the functional importance of the detected variation, we mapped the PSSs on the modelled 3D structure of the duck RIG-I protein (Figure 4), determining their accessibility to ligands and site-specific functional annotation (Figure S5). The majority of PSSs were located at the molecular surface of the protein and distributed within the ligase domains: five in the HEL1, four in the HEL2 and CTD domain. Only two positions were located in close topological proximity to the known functionally relevant sites or were previously identified as directly being the functional sites: the residue 859 is located close to the RNA ligand binding site, and the residue 668 represents a phosphorylation site. Eight out of the 22 PSSs detected in our dataset have been previously identified also by other studies as being under positive selection (Figure S5).

**Figure 5:**
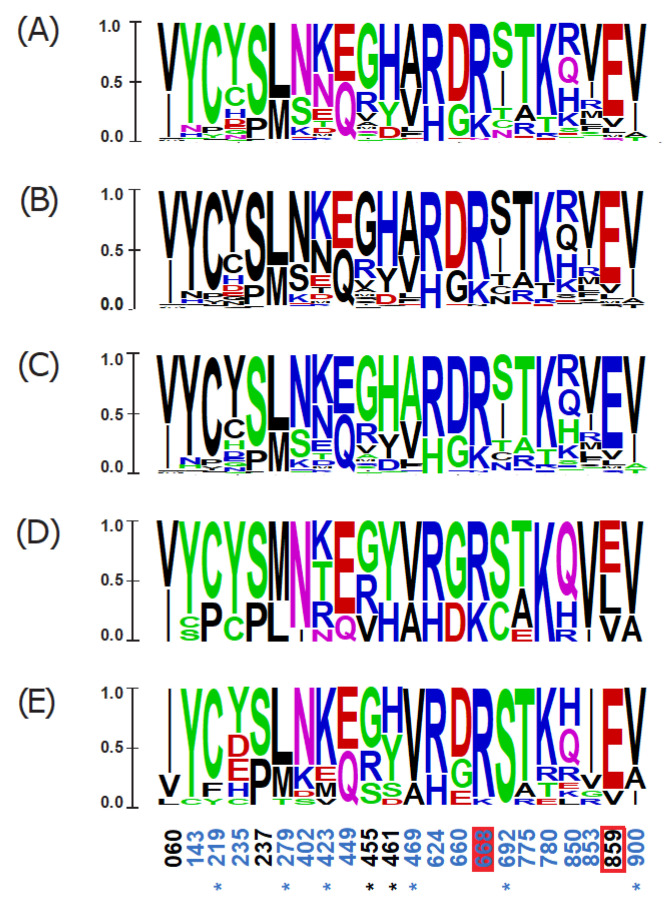
Physicochemical properties of the positively selected sites in avian RIG-I gene. Letter diagrams show the proportion of specific amino acids in RIG-I in all species (**A**–**C**), in nine species with the disrupted MDA5 (**D**), and in fifteen closely related species with functional MDA5 (**E**). Size of a letter indicates the frequency of a particular amino acid within the sequence alignment. Amino acid substitutions are coloured according to their physicochemical properties. For rows (**A**,**D**,**E**) acidic amino acids are in red, basic in blue, neutral in purple, polar in green and hydrophobic in black. For PSS in all species, charge (negative in red and positive in blue; (**B**) and hydrophobicity (hydrophilic in blue, hydrophobic in black, and neutral in green; (**C**) are also shown separately. Surface accessible amino acids assessed from the “open” model are in blue and PSSs identified also in other studies are labelled with an asterisk. PSSs in close proximity to functional sites, and consistent with functional sites are highlighted with a red rectangle, and with filled red box, respectively. As closely related species with functional MDA5, the following species were selected: related to Ciconiiformes—*Nipponia nippon*, *Phalacrocorax pelagicus*, *Nannopterum brasilianus*, *Calonectris borealis*, *Hydrobates tethys*, *Egretta garzetta*; related to Gruiformes—*Phoenicopterus ruber*, *Recurvirostra avosetta*, *Himantopus himantopus*, *Scolopax mira*, *Calidris pugnax*, *Limosa lapponica*, *Sterna hirundo*, *Alca torda* and *Uria lomvia*. Duck numbering is adopted (RIG-I: GenBank ID EU363349.1).

To further inspect amino acid sequence variability at PSSs that could be linked to the *MDA5* loss, we compared the variant-frequency diagrams between (i) all the species tested, (ii) the nine species missing a functional *MDA5* gene, and (iii) 15 selected species that are closely related to the *MDA5*-missing taxa (Figure 5). Consistent with our previous results, we did not find any RIG-I PSS that would show an overrepresentation of any of the specific substitutions in the *MDA5*-lacking species. Together, our results do not support the hypothesis of compensatory evolution in *RIG-I* driven by the *MDA5* loss.

## 4. Discussion

In this study, we report the genetic inactivation of the *MDA5* virus sensor in two avian clades: the storks (Ciconiiformes) and the rallids (Gruiformes). This represents the first documented evolutionary loss of this gene in birds. Our results are based on in silico analysis of publicly available avian genomic and transcriptomic data. However, we provide strong evidence that our results cannot be explained by errors in sequencing or genomic contig assembly. The pattern of inactivating mutations is consistent with the phylogenetic hypothesis of two independent initial inactivation events followed by random accumulation of subsequent mutations. The sharing of inactivating mutations (stops, indels, larger deletions) among multiple species within each avian lineage provides strong support for this scenario (Figure 2). Furthermore, we described a relaxed negative selection in the genetic sequences of *MDA5*-deficient species (Figure 3), which is also consistent with the functional disruption of the *MDA5* gene. We did not observe any indication of duplication of *MDA5* or *RIG-I* genes in our homology-based searches. Finally, in larger genomic contigs that allowed for analysis of syntenic gene order, we observed some of the closest neighboring genes to chicken *MDA5* (data not shown). This provides an independent line of evidence pointing to orthology of avian *MDA5* genes analyzed.

In contrast to our prediction, we did not find any clear evidence of compensatory selection in the RIG-I sensor in the *MDA5*-deficient avian species. Our branch-site tests of positive selection were significant only for the entire avian phylogeny; the *MDA5*-deficient branches did not score as significant in the tests (Table 1). One reason for this outcome could be the low number of *MDA5*-deficient avian species available for the analysis. The high total number of analyzed species allowed us to describe and annotate the highest number of PSSs in avian RIG-I detected so far. Yet, the comparison of PSSs between the *MDA5*-positive and *MDA5*-negative species also did not yield any consistent specific differences for the *MDA5*-negative species.

While species-specific repertoires of the pattern recognition receptors are known and well documented in the genomic databases, gene loss events have only been rarely described in these genes. The loss of *MDA5* was described only once in vertebrates, in the common ancestor of three species of pangolins [43]. Further evolutionary gene losses in the RLR family, besides the ones described above in birds, include *RIG-I* inactivation in tree shrews [44]. Other notable cases of innate immune genes lost include the loss of *Mx1* and *Mx2* in whales [45], the loss of *PYHIN* genes in bats [46], and the loss of Toll-like receptor 5 (*TLR5*) in several bird [47,48] and mammalian lineages [49].

The reasons and evolutionary consequences of such events are not fully understood. However, there are two general scenarios suggested to explain non-detrimental outcomes of such pseudogenization events: (i) the loss of an infection sensor is accompanied by an acquired tolerance to particular pathogens and (ii) the missing sensor gene is functionally replaced by a related gene or by another sensing pathway. Consistent with the second scenario, the tree shrew MDA5 was shown to be able to detect the Sendai virus, otherwise considered to be a RIG-I agonist [44]. In contrast, in the chicken a decade-long intensive research did not bring any conclusive understanding of the extent of compensatory changes that occurred in chicken *MDA5* after the *RIG-I* loss [16,18,19,50,51,52,53]. Our current work provides a complementary model in which the other RLR sensor (here MDA5) is lost in birds. Future experimental studies will determine whether, for example, stork RIG-I shows an expanded ability to recognize viral RNA structures. Our findings provide further support for the dynamic evolution of RLRs and propose new questions about the redundancy and flexibility of the RNA-virus-sensing apparatus in vertebrates.

## Figures and Tables

**Table 1:** Significance of positive selection pressure acting on avian RIG-I.

Clade	Test of Positive Selection (PAML) ^a^		
	dN/dS (%) ^b^	LRT	*p* value ^c^
whole phylogeny	2.4 (4.5%)	194.955	0.0000 ***
MDA5-missing branches	2.2 (1.8%)	3.332	0.0680

^a^ Branch-site test of positive selection in codeml program of PAML package. ^b^ dN/dS ratio estimate of the class of codons under positive selection with the percentage of codons falling into this class designated in parentheses. ^c^ *p* values calculated from likelihood ratio test (LRT) statistics; level of significance is expressed by asterisks (*p* < 0.0001).

## Data Availability

Data is contained within the article or Supplementary Material.

## Supplementary Materials

The following are available online at https://www.mdpi.com/article/10.3390/v13112131/s1, Figure S1: *MDA5* and *RIG-I* coding sequences of avian species analyzed in our study (Fasta format), Figure S2: accession numbers of genomic contigs and SRA datasets used to identify avian *MDA5* genes (Excel xlsx format), Figure S3: Nucleotide alignment of avian *MDA5* coding sequences, where nonfunctional orthologs were manually corrected (Fasta format), Figure S4: Nucleotide alignment of avian *RIG-I* coding sequences (Fasta format), Figure S5: Table of annotated PSSs identified in avian *RIG-I* gene (Excel xlsx format).
